# Changes in faecal haemoglobin values over sequential rounds of faecal immunochemical tests (FIT) in a surveillance population

**DOI:** 10.1136/bmjgast-2024-001651

**Published:** 2025-02-11

**Authors:** Gemma Mortell, Kate Wooldrage, Gwen A Murphy, Amanda J Cross

**Affiliations:** 1 Department of Surgery and Cancer, Imperial College London - St Mary's Campus, London, UK

**Keywords:** CANCER EPIDEMIOLOGY, COLORECTAL CANCER, COLORECTAL ADENOMAS, COLORECTAL CANCER SCREENING

## Abstract

**Objective:**

Colorectal cancer (CRC) screening enables resection of polyp precursor lesions, preventing cancer or detecting it earlier. Post-polypectomy, people can remain at increased CRC risk, prompting surveillance colonoscopy. Less invasive faecal immunochemical tests (FIT) could reduce the burden of surveillance colonoscopy. We investigated whether changes in FIT values over multiple rounds were associated with advanced colorectal neoplasia (ACN) detection.

**Methods:**

A cohort of men and women aged 60–72 years deemed intermediate risk by the 2002 UK adenoma surveillance guidelines and scheduled for three yearly colonoscopies were recruited (January 2012–December 2013) within the English Bowel Cancer Screening Programme and offered a quantitative FIT at 1, 2 and 3 years post-baseline colonoscopy for a prospective analysis within a diagnostic accuracy study. Participants positive (≥40 µg haemoglobin/g faeces) at 1 year or 2 years were offered early colonoscopy and excluded, otherwise, colonoscopy was offered at 3 years. Only those who completed three FIT rounds and attended the 3-year colonoscopy were included. Participants were grouped based on changes between FIT rounds, with changes defined as absolute differences ≥4 µg/g, and positivity at round 3.

**Results:**

Among 4412 participants, the largest group (n=2773) was the no change category, which had the lowest ACN detection rate (4.7%, 95% CI: 3.9 to 5.5). The serial increase group with a positive round 3 value (n=46) had the highest ACN detection rate (32.6%, 95% CI: 19.5 to 48.0).

**Conclusion:**

No change in FIT result across multiple rounds was associated with a low ACN detection rate, while a serial increase was associated with higher ACN detection rates. Further research should consider if sequential rounds of FIT could be used for stratifying individual risk.

**WHAT IS ALREADY KNOWN ON THIS TOPIC**

- Post-polypectomy surveillance places a significant burden on patients and can cause challenges related to resource allocation for endoscopy services.
- Faecal immunochemical tests (FIT) are widely used to screen for colorectal cancer and could be used in a surveillance capacity over multiple rounds of testing but little data exists on this.

**WHAT THIS STUDY ADDS**

- This study examines the variability in faecal haemoglobin over three annual rounds of FIT testing in a surveillance population.
- Participants with no change in faecal haemoglobin across all rounds of FIT had the lowest advanced colorectal neoplasia (ACN) detection rate of 4.7% (95% CI: 3.9 to 5.5) and the highest detection rate was observed in those with a serial increase in FIT value from round 1 to round 2, but the FIT value remained below the positivity threshold until round 3, when it reached the positivity threshold (ACN detection rate: 32.6%, 95% CI: 19.5 to 48.0).

**HOW THIS STUDY MIGHT AFFECT RESEARCH, PRACTICE OR POLICY**

- This research suggests that no faecal haemoglobin, rather than decreasing levels, might be preferable to inform on the lowest-risk group and that sequential FITs could be used to inform surveillance guidelines.

## Introduction

 Colorectal cancer (CRC) remains a major health burden with high mortality globally. In 2020, more than 1.9 million new CRC cases and 935 000 deaths due to this disease were estimated to occur, representing about one in 10 cancer cases and deaths.^1^ Despite this, CRC remains one of the most amenable cancers to screening, with a range of techniques implemented worldwide,^2^ allowing both early detection and prevention via removal of polyp precursor lesions.

The British Society of Gastroenterology and the Association of Coloproctology of Great Britain and Ireland recommend colonoscopy surveillance for those who are deemed to remain at high risk following polypectomy.^3^ Post-polypectomy surveillance places significant demands on endoscopic services and was estimated to account for approximately 20% of all colonoscopies in the UK.^4^ While colonoscopy remains an important surveillance modality, it should be directed towards people where the expected benefits outweigh potential harms. Faecal immunochemical tests (FIT) are commonly used for screening by estimating faecal haemoglobin levels in stool samples. Screening programme use of quantitative FIT involves the selection of a faecal haemoglobin threshold level for positivity, which can be adjusted for the desired clinical sensitivity and specificity based on the resources available. FIT can also be used at frequent intervals, with multiple rounds potentially improving test performance. While these benefits have resulted in FIT being integrated into many screening programmes, few studies have examined the role of FIT as a cheaper and less invasive modality for surveillance.

Little data exists regarding whether increases or decreases in faecal haemoglobin level over multiple rounds of FIT are associated with the detection of advanced adenomas or CRC. To address this, we examined the variation in faecal haemoglobin levels over three annual rounds of FIT among individuals recommended surveillance.

## Methods

### Study design and participants

The ‘FIT for Follow-up’ study was a diagnostic accuracy study designed to investigate the performance of FIT for surveillance.^5^ The London (City and East) Research Ethics Committee approved the study in 2011 (reference 11/LO/0326). Between January 2012 and December 2013, 8009 eligible individuals (aged 60–72 years) categorised as intermediate risk (UK 2002 adenoma surveillance guidelines^6^) following colonoscopy as a result of a positive guaiac faecal occult blood test (gFOBT) within the English Bowel Cancer Screening Programme (BCSP) were invited to take part in the study. Intermediate-risk participants included those with three to four small adenomas or one or two adenomas with at least one adenoma ≥1 cm in size^6^; these participants were scheduled to undergo a 3-yearly surveillance colonoscopy. At the time, the BCSP invited those aged 60–74 years to take part in gFOBT screening, but we excluded those aged 73–74 years from this study, as they would have been ≥75 years at the end of the 3 years and not eligible for surveillance colonoscopy. Invitations, FIT kits and written consent forms for the study were sent by the BCSP Southern Hub. To avoid participants being over-investigated, participants were excluded if they had received more than one baseline colonoscopy.

**Figure 1:**
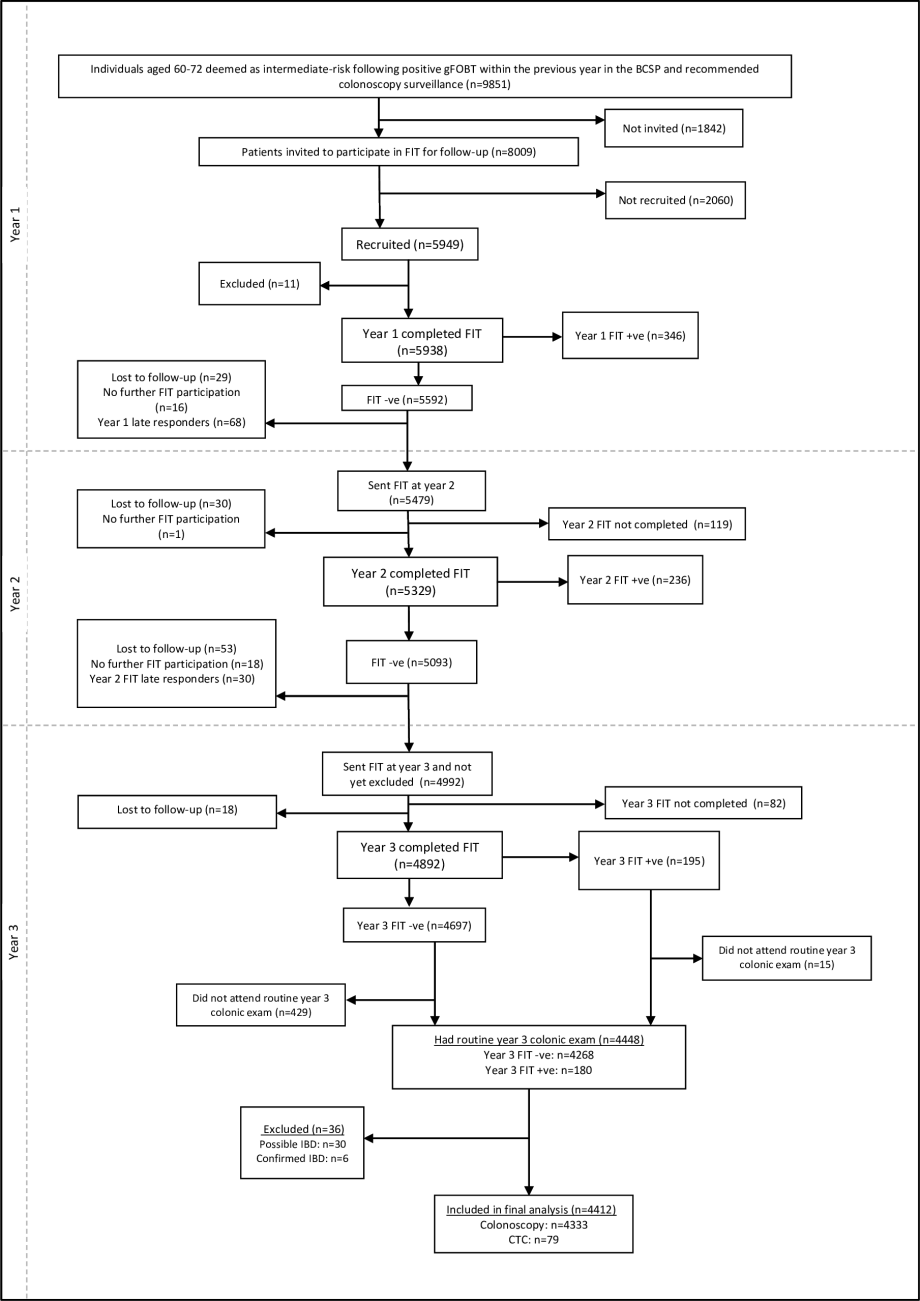
Participant flow diagram from invitation to year 3 colonic examination. CTC, CT colonography; FIT, faecal immunochemical test; gFOBT, guaiac faecal occult blood test; IBD, inflammatory bowel disease; +ve, positive; –ve, negative.

In total, 5949 people were recruited into the study, provided written informed consent (figure 1) and completed the first FIT (OC-AUTO Sampling Bottle 3, Eiken Chemical Co., Ltd, Japan). The study involved three rounds of FIT, conducted at 1, 2 and 3 years post-baseline colonoscopy. Only participants who returned a round 1 FIT were invited to subsequent rounds. Those who tested positive (≥40 µg haemoglobin/g faeces) in rounds 1 or 2 were offered an early colonoscopy and were not invited to complete further FITs. Participants in round 3 were scheduled for colonoscopy 3 years post-baseline, in line with the UK recommendations at the time for those deemed as intermediate risk.

To be included in this prospective analysis, participants had to have returned all three rounds of FIT and attended the year 3 colonic examination. Participants with a positive FIT result at round 1 or round 2 were referred for early colonoscopy and excluded from this analysis. Participants with poor or incomplete year 3 colonic examinations and those with confirmed or possible inflammatory bowel disease were also excluded from this analysis.

The outcomes measured were (i) advanced adenomas (defined as adenomas ≥10 mm, with villous or tubulovillous histology, or with high-grade dysplasia), (ii) CRC and (iii) advanced colorectal neoplasia (ACN), which included both advanced adenomas and CRC. We defined CRC sites by the International Classification of Diseases 10th revision, using codes C18–C20 and CRCs of all morphologies were included.

The FIT positivity threshold used in these analyses was 40 µg/g (200 ng/mL). The analyses were based on a number of assumptions as per the original study^5^: that colonoscopy detects all ACN and that any ACN detected at year 1, 2 or 3 was present at year 1 and would have remained present and unmodified, in the absence of colonic examination, to year 3. We assumed that the same ACN would be detected in each participant regardless of the year colonoscopy was performed. The lower limit of detection is the lowest concentration at which faecal haemoglobin can be detected 95% of the time (10 µg/g for the OC-Sensor DIANA analyser used by the BCSP [Eiken Chemical Co., Ltd, Japan])^7 8^ and the lower limit of quantification refers to the lowest amount of faecal haemoglobin that can be reliably measured^9^ (4 µg/g for the OC-Sensor DIANA).^10^ However, the Guildford Medical Device Evaluation Centre published an evaluation of analytical sensitivity tests on a variety of devices, which determined that the OC-Sensor DIANA analyser had a lower limit of detection (3.8 µg/g) lower than that quoted by the manufacturer (10 µg/g).^7^ We therefore used 4 µg/g (20 ng/mL) as the lower limit of detection and thus recoded any FIT values that were less than 4 µg/g (20 ng/mL) to zero for all subsequent calculations. The reporting of this study conforms to the Strengthening the Reporting of Observational Studies in Epidemiology statement (online supplemental file A).

### Patient and public involvement

Some patient and public representatives had no prior history of CRC or colonoscopy and some were actively undergoing post-polypectomy surveillance. These representatives were invited to collaborate on designing patient materials and questionnaires and having their perspectives represented as members of the independent steering committee. Furthermore, a panel of representatives gave feedback on developing study materials (eg, flyer, letter, consent form, baseline questionnaire, FIT kit instructions, participant information sheet). Their insights played a key role in developing this content and our approach to contacting potential participants. Other service users were interviewed to understand the likely acceptability of FIT as a CRC surveillance tool and another discussion group was hosted to discuss the practicalities of completing a FIT. A number of important and effective modifications to the study materials and patient management system were made as a result of their input.

### Statistical analysis

For the purpose of this analysis, FIT values and the differences in results between rounds were calculated using the FIT values recorded in ng/mL of buffer, as this is how the instrument reported the numeric results, rather than µg/g, which is used in clinical reporting. We calculated the difference in FIT results between round 1 and round 2 and between round 2 and round 3. Considering the instrument’s lower limit of detection and lower limit of quantification and that we recoded FIT values <4 µg/g (20 ng/mL) to zero, we defined that a change in FIT value occurred between rounds if the absolute value of the difference in FIT result was ≥4 µg/g (20 ng/mL).

Participants were classed into 12 groups based on the changes seen in their FIT results between rounds. Participants that had no differences between rounds were categorised as the no change group. Participants with serial increases were categorised into two groups: a serial increase across all three rounds where no FIT result passed the positivity threshold of 40 µg/g (200 ng/mL); or a serial increase across all three rounds where the FIT result was below the positivity threshold at round 1 and round 2 but above the positivity threshold at round 3. Participants with serial decreases were not categorised further as no FIT result could pass the positivity threshold, as the results were decreasing consistently. We categorised participants with variable changes into eight groups based on the combinations of increase, decrease or no change seen between rounds and whether the FIT result at round 3 passed the positivity threshold. We calculated ACN detection rates by dividing the number of participants with ACN detected by the total number of participants within each group and estimated exact 95% CI.

We performed a sensitivity analysis where an absolute difference in FIT value between rounds of >0 µg/g (0 ng/mL), rather than ≥4 µg/g (20 ng/mL), was considered a change. We report and discuss results in micrograms per gram (µg/g) to facilitate the comparison of results across studies, as was recommended by the Expert Working Group (EWG) on FIT for Screening, Colorectal Cancer Screening Committee, World Endoscopy Organization.^11^ Analyses were performed in Stata/IC 16 (V.16.1) (College Station, TX, USA: StataCorp LLC).

## Results

Of the 5949 participants recruited, 4892 completed all three rounds of FIT, of whom 4448 had a year 3 colonic examination. We excluded 36 participants with possible or confirmed inflammatory bowel disease, leaving 4412 for this analysis (figure 1). For the end of study colonic examination, 79 had a CT colonography (CTC) and 4333 had a colonoscopy. The study population was 65.2% male and 50.9% were aged >65 years old. In total, 312 (7.1%, 95% CI: 6.3 to 7.9) participants were diagnosed with ACN, of whom six were diagnosed with CRC (table 1).

**Table 1:** Baseline characteristics of study population and detection of advanced colorectal neoplasia and colorectal cancer (n=4412)

	Study population	Advanced colorectal neoplasia detected	Colorectal cancer detected
Total, n (%)	4412 (100)	312 (7.1)	6 (0.14)
Sex, n (%)			
Male	2878 (65.2)	209 (7.3)	2 (0.07)
Females	1534 (34.8)	103 (6.7)	4 (0.26)
Age at invite, years, n (%)			
≤65	2168 (49.1)	148 (6.8)	4 (0.18)
>65	2244 (50.9)	164 (7.3)	2 (0.09)

When the participants were categorised into the 12 groups according to the serial changes in their FIT values across all three rounds, the largest group (n=2773, 62.9%) was those who experienced no change in FIT value across all three rounds (table 2); this group had the lowest ACN detection rate (4.7%, 95% CI: 3.9 to 5.5) of all of the groups. Further analysis showed that the vast majority (99.5%) of participants in this group had FIT values of zero across all three rounds and only 14 participants in this group (0.5%) had non-zero FIT values that remained unchanged (online supplemental table 1).

**Table 2:** Serial changes groups and detection of advanced colorectal neoplasia (n=4412)

	Serial changes group*	Total, n(%)	No ACN detected, n(%)	ACN detected, n% (95% CI)
**Total**		4412 (100)	4100 (92.9)	3127.1 (6.3 to 7.9)
1.	Serial increase above threshold (R2↑† – positive change, below threshold), (R3↑† – positive change, above threshold)	46 (1.0)	31 (67.4)	15‡32.6 (19.5 to 48.0)
2.	Serial increase below threshold (R2↑† – positive change, below threshold), (R3↑† – positive change, below threshold)	58 (1.3)	48 (82.8)	10‡17.2 (8.6 to 29.4)
3.	Variable changes – R2↑†, R3↓§	435 (9.9)	392 (90.1)	43‡9.9 (7.2 to 13.1)
4.	Variable changes – R2↑†, R3↔¶	59 (1.3)	52 (88.1)	711.9 (4.9 to 22.9)
5.	Variable changes – R2↓§, R3↑† (positive change, above threshold)	41 (0.9)	30 (73.2)	1126.8 (14.2 to 43.0)
6.	Variable changes – R2↓§, R3↑† (positive change, below threshold)	126 (2.9)	105 (83.3)	2116.7 (10.6 to 24.3)
7.	Serial decrease ↓§ (serial decreases over all 3 rounds)	50 (1.1)	45 (90.0)	510.0 (3.3 to 21.8)
8.	Variable changes – R2↓§, R3↔¶	320 (7.3)	293 (91.6)	278.4 (5.6 to 12.0)
9.	Variable changes – R2↔¶, R3↑† (positive change, above threshold)	91 (2.0)	80 (87.9)	1112.1 (6.2 to 20.6)
10.	Variable changes – R2↔¶, R3↑† (positive change, below threshold)	367 (8.3)	339 (92.4)	287.6 (5.1 to 10.8)
11.	Variable changes – R2↔¶, R3↓§	46 (1.0)	42 (91.3)	48.7 (2.4 to 20.8)
12.	No change (FIT values the same across all rounds)	2773 (62.9)	2643 (95.3)	130‡4.7 (3.9 to 5.5)

* A change is defined as the absolute value of the difference in FIT values between rounds being ≥4 µg/g (20 ng/ml ng/mL). The positivity threshold is defined as 40 µg/g µg/g (200 ng/ml ng/mL).

† ­­↑ Increase in FIT value from previous round.

‡ Six participants were diagnosed with colorectal cancer: two in group 1, one in group 2, one in group 3 and two in group 12.

§ ↓ Decrease in FIT value from previous round.

¶ ↔ No change in FIT value between rounds.

ACN, advanced colorectal neoplasia; FIT, faecal immunochemical test; R2, round 2; R3, round 3

The next largest group was those with variable changes with a round 2 increase and round 3 decrease (n=435, 9.9%), then those with variable changes with no change at round 2 and an increase that remained below the positivity threshold at round 3 (n=367, 8.3%), and then those with variable changes with a decrease at round 2 and no change at round 3 (n=320, 7.3%); the ACN detection rate in these three groups was similar (9.9%, 95% CI: 7.2 to 13.1; 7.6%, 95% CI: 5.1 to 10.8; and 8.4%, 95% CI: 5.6 to 12.0, respectively). All other groups made up <3% of participants.

The group with the highest ACN detection rate was those with a serial increase above the threshold, with an increased FIT value from round 1 to round 2 but the FIT value remained below the positivity threshold until round 3, when it reached above the positivity threshold (table 2). In this group, 15 out of 46 participants had ACN detected (ACN detection rate: 32.6%, 95% CI: 19.5 to 48.0). Although those with a serial increase in FIT values that remained below the positivity threshold at all three rounds (n=58, 1.3%) had a relatively high ACN detection rate (17.2%, 95% CI: 8.6 to 29.4), the group with the second highest rate of ACN detection was a group with variable changes, where the FIT value decreased from round 1 to round 2 and increased from round 2 to round 3 to above the positivity threshold. Here, 11 out of 41 participants in this group had ACN detected (ACN detection rate: 26.8%, 95% CI: 14.2 to 43.0). Similarly, in the group where the FIT value decreased from round 1 to round 2 and increased from round 2 to round 3 but stayed below the positivity threshold, 21 out of 126 participants had ACN detected (ACN detection rate: 16.7%, 95% CI: 10.6 to 24.3).

**Figure 2:**
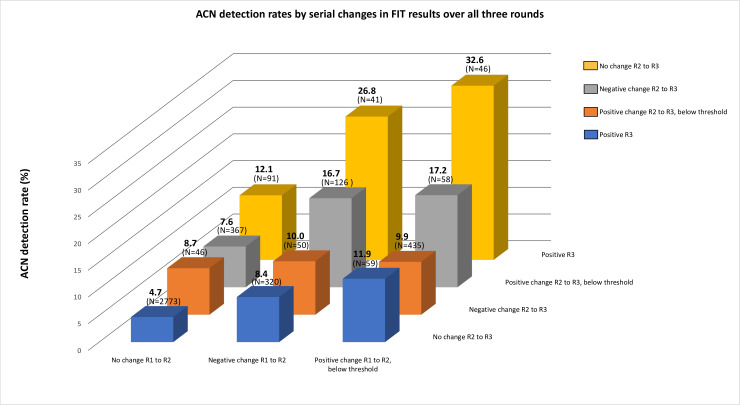
Advanced colorectal neoplasia (ACN) detection rates by serial change in faecal haemoglobin results over all three rounds of faecal immunochemical test (FIT) (n=4412). R2, round 2; R3, round 3.

Figure 2 shows graphically that those who were FIT positive at round 3 after having a negative change or a positive change between rounds 1 and 2 had the highest ACN detection rates. Having a positive change that was below the positivity threshold between rounds 2 and 3 also resulted in relatively high ACN detection rates when there was either a negative or positive change in FIT values between rounds 1 and 2. These four groups that displayed the highest ACN detection rates all had an increase in FIT value from round 2 to round 3 and all participants had at least one prior non-zero FIT result. The three groups with the lowest ACN detection rates (groups 8, 10 and 12) had the highest rates (≥94%) of having at least two zero FIT values across rounds (online supplemental table 1).

The results showed that a positive change (below the positivity threshold) between rounds 1 and 2 did not necessarily result in a high ACN detection rate; detection rates were 11.9% and 9.9% if there was no change or a negative change at round 3, respectively. Participants who had their FIT value increase further after an increase at round 2 had high ACN detection rates (groups 1 and 2, detection rates of 32.6% and 17.2%, respectively).

When we performed a sensitivity analysis to investigate if our definition of a change in FIT value was affecting our results, we found that expanding the definition of a change to include absolute differences of values between >0 µg/g and <4 µg/g (1–19 ng/mL) between rounds altered the group classification for only 3.6% of participants and our results were not materially changed (online supplemental table 2).

## Discussion

We examined whether variability in faecal haemoglobin level over sequential rounds of FIT was associated with ACN detection rates in a post-polypectomy surveillance population. FIT is used in national screening programmes by selecting a predefined ‘positivity threshold’; people testing above this will normally be referred for colonoscopy for further examination. FIT is not routinely used in post-polypectomy surveillance due to a lack of data on its use in this capacity.

Faecal haemoglobin concentration has been associated with the severity of colorectal neoplasia and lesion size in screening populations.^12 13^ Digby *et al* reported higher faecal haemoglobin concentrations at a single round of FIT in those with high-risk adenomas detected (defined as ≥3 adenomas or any adenoma with a diameter ≥10 mm) compared with those with low-risk adenomas detected (defined as <3 adenomas of diameter <10 mm) in a population from the Scottish Bowel Screening Programme.^12^ In our study, the group with the lowest ACN detection rate was participants with sequential FIT values of zero (0–<4 µg/g) over three rounds. Similarly, Meester *et al* found that in a screening population with three rounds of FIT administered at two yearly intervals, participants with consecutive FIT values of 0 µg/g had a lower risk of ACN.^14^ Our results suggest that monitoring trends in faecal haemoglobin over consecutive rounds could be used to predict ACN detection rates in a post-polypectomy population, particularly in participants showing a consistent, unchanged FIT result of zero, which accounted for 62.9% of our population.

A study by Grobbe *et al* investigated the association between baseline faecal haemoglobin concentration and ACN development over four rounds of biennial screening in a population of FIT-negative participants (baseline FIT value below a cut-off value of 10 µg/g faeces). They reported that participants with baseline results of 8–10 µg/g had an increased ACN risk compared with those with baseline concentrations of 0 μg/g.^15^ Another group investigated the role of faecal haemoglobin concentration in predicting ACN risk in a FIT-negative screening population in Italy. Over two consecutive rounds of FIT given 2 years apart, participants with cumulative faecal haemoglobin concentrations of ≥20 µg/g faeces showed a higher risk of ACN being detected at subsequent rounds, while participants with undetectable faecal haemoglobin had a lower risk of ACN being detected at subsequent rounds.^16^

A study examining a FIT-negative screening population, defined as a FIT result <20 µg/g, grouped the negative FIT results into three categories: non-detectable FIT (0–3.8 µg/g), low negative FIT (3.9–9.9 µg/g) and high negative FIT (10–19.9 µg/g). This study found that participants who had two consecutive negative FITs, 2 years apart, that were above the limit of detection and subcategorised as low negative FIT or high negative FIT, were at greater risk of being diagnosed with ACN or incident cancer compared with those categorised as non-detectable FIT.^17^ Similarly, this study found that the probability of being diagnosed with ACN or incident cancer rose with increasing values of FIT, i.e. participants with two consecutive high negative FIT results were at the greatest risk.

While these previous studies were conducted in screening populations rather than surveillance populations and most used a positivity threshold of 20 µg/g, our findings show similar trends of multiple, low faecal haemoglobin concentrations that fall below the positivity cut-off (40 µg/g) over repeated rounds of FIT, particularly multiple values that are zero, being associated with lower ACN detection rates in a surveillance population. One study has examined FIT in a surveillance programme and found that multiple rounds of negative FIT were associated with a low risk of advanced neoplasia.^18^ All these results, including our own, suggest that further risk stratification can be informative in a FIT-negative population, as lower ACN rates were observed in those with results close to zero or those with multiple zero values. This indicates there is potential to categorise ACN risk even more precisely. To our knowledge, we are one of the first to examine changes in faecal haemoglobin concentrations over multiple rounds of annual FIT and ACN detection rates in a surveillance population.

We found that the lowest rate of ACN detection (4.7%) was in the group that had no change across all three rounds and the groups with the three lowest detection rates (4.7%–8.4%) had the highest rates of having at least two zero FIT values. The highest ACN detection rates (16.7%–32.6%) occurred in the groups where all participants had a recent increase in FIT value and at least one prior non-zero value. These results suggest that multiple rounds of FIT could be predictive in terms of ACN detection.

The groups with the highest ACN detection rates were two of the three subgroups that were FIT positive at round 3—those with a serial increase above the threshold and those whose FIT result decreased from round 1 to round 2 but increased to above the positivity threshold at round 3. The group with the third highest ACN detection rates was those with a serial increase over all three rounds but did not pass the positivity threshold. While this reiterates previous findings that a single positive FIT result is associated with increased ACN detection, these results could further support the theory that an increase in faecal haemoglobin over multiple rounds is associated with increased ACN risk. Chen *et al* examined FIT-negative participants in a screening population in Taiwan and reported that those with higher initial baseline faecal haemoglobin concentrations showed increased incidence of adenoma and CRC and that incidence increased as faecal haemoglobin concentration increased. They also reported that the higher the initial faecal haemoglobin concentration, the higher the likelihood of developing advanced adenomas.^19^

Our results open a discussion about the use of multiple rounds of FIT testing in surveillance. There may be value in considering a baseline FIT result at round 1 and comparing quantitative changes in faecal haemoglobin at subsequent rounds. We showed that 62.9% of our population had no change in FIT value across three rounds and had very low ACN detection rates, suggesting that colonoscopy surveillance in these people may not be necessary, which could reduce the burden on endoscopy services and eliminate some of the risks associated with colonoscopy.

Further research should explore the benefit of further stratifying people already under surveillance into risk groups. Chen *et al* suggested categorising FIT-negative people in a screening context into low, intermediate, high and extremely high-risk groups using faecal haemoglobin concentrations (converted from the reported scale of ng/g to μg/g) of 0.2–3.8, 4–7.8, 8–15.8, and 16–19.8 µg/g, respectively.^19^ Similarly, our results highlight the potential use of categorising people based on the types of serial changes in their faecal haemoglobin levels over multiple rounds. Our data suggest that having no detectable faecal haemoglobin rather than decreasing levels might be preferable to inform on the lowest-risk group. The serial decrease group in our study was small (n=50), so further research with a larger sample size would be needed to draw firm conclusions about the significance of a decreased trend in faecal haemoglobin. Furthermore, additional studies investigating all 12 categories could be informative to confirm which categories could be collapsed.

There are a number of difficulties in implementing quantitative FIT-informed surveillance in practice. FIT national screening programmes and investigative studies^11^ generally only report whether the person is FIT positive or FIT negative rather than the numerical value of faecal haemoglobin. Furthermore, it is difficult to generalise results considering the variability in (limit of quantification, limit of detection) instruments from different manufacturers and the variability in units of measurement used. The EWG recommends that all manufacturers, suppliers and users of FIT report results in μg haemoglobin/g faeces to enable universal comprehension and transferability of data across FIT systems.^11^ For our analysis, we used the unit ng/mL during the calculations as it has been proposed that low faecal haemoglobin concentrations should be reported as whole numbers. We discuss the results in µg/g to comply with EWG recommendations.

Currently, there is no consensus about what constitutes a ‘change’ in faecal haemoglobin concentration between rounds. We defined change as an absolute difference between FIT values that was ≥20 ng/mL (≥4 µg haemoglobin/g faeces) and also recoded individual FIT values below this to zero, in line with the OC-Sensor DIANA’s lower limit of detection.^7^ Caution should be used in the interpretation of these absolute values because different instruments with different limits of detection may lead to varying definitions of a ‘meaningful change’ in faecal haemoglobin concentration between rounds.

Strengths of our study include that, to our knowledge, this is the first study to evaluate changes in FIT values across multiple rounds in a post-polypectomy surveillance population. Our population is a relatively large sample of clearly defined post-polypectomy participants and each participant included was given a colonoscopy or CTC, giving us a clearly defined endpoint. Another strength is that the original study had a high rate of FIT return, with initial FIT return of 74% and subsequent FIT return of 97% for round 2 and round 3.^5^

A limitation of this study is that we could not stratify participants by age or sex due to the relatively small size. The small number of CRCs meant we had to assess ACN detection rates as the outcome of interest rather than advanced adenomas and cancers separately. Furthermore, we could not examine beyond 3 years of follow-up and, given that colorectal neoplasia can take several years to develop, future work with longer follow-up is needed to validate our findings. While we did exclude those with suspected or confirmed inflammatory bowel disease from this analysis, there likely remained participants who were undiagnosed. In addition, the presence of haemorrhoids could influence FIT values, although there are conflicting reports, with one study finding they were not associated with false positive FIT values^20^ and another study reporting they were associated with higher faecal haemoglobin concentrations.^21^

## Conclusion

To our knowledge, this is one of the first investigations into sequential faecal haemoglobin concentrations and ACN risk in a post-polypectomy surveillance population. The highest rates of ACN detected were observed in two groups that experienced a positive round 3 FIT result. The lowest ACN detection rate was observed in participants that had no change in FIT value across all three rounds. These results suggest there is potential in using quantitative data from multiple rounds of FIT to predict ACN risk and that reporting the numerical FIT result could inform clinical decision making. Future work is needed to validate these results to ensure they are reproducible using different instruments with different limits of detection and limits of quantification.

## supplementary material

10.1136/bmjgast-2024-001651 online supplemental file 1

10.1136/bmjgast-2024-001651 online supplemental table 1

## Data Availability

Data are available upon reasonable request.
