# Supplementary material for: Changes in faecal haemoglobin values over sequential rounds of faecal immunochemical tests (FIT) in a surveillance population
Source: BMJ Open Gastroenterol. 2025 Feb 11;12(1):e001651. doi: 10.1136/bmjgast-2024-001651 (PMC11843008; doi:10.1136/bmjgast-2024-001651)
Supplement: online supplemental table 1 [file bmjgast-12-1-s002.pdf]

Supplementary Table 1

Supplementary table 1. Advanced colorectal neoplasia (ACN) detection rate and number of zero faecal immunochemical test results by serial change groups (sorted in order of descending ACN detection rate).

Group number	Serial changes groups* sorted in descending order of ACN detection	Total N=4412	ACN detection rate (%)	Three, n (%)	Two, n (%)	≤ one zero (i.e. ≥ two non-zero), n (%)
1.	Serial increase above threshold (R2↑† – positive change, below threshold), (R3↑† – positive change, above threshold)	46‡	32.6	0 (0%)	0 (0%)	46 (100%)
5.	Variable changes – R2↓§, R3↑† (positive change, above threshold)	41	26.8	0 (0%)	0 (0%)	41 (100%)
2.	Serial increase below threshold (R2↑† – positive change, below threshold), (R3↑† – positive change, below threshold)	58‡	17.2	0 (0%)	0 (0%)	58 (100%)
6.	Variable changes – R2↓§, R3↑† (positive change, below threshold)	126	16.7	0 (0%)	0 (0%)	126 (100%)
9.	Variable changes – R2↔¶, R3↑† (positive change, above threshold)	91	12.1	0 (0%)	80 (88%)	11 (12%)
4.	Variable changes – R2↑†, R3↔¶	59	11.9	0 (0%)	0 (0%)	59 (100%)
7.	Serial decrease↓§ (serial decreases over all 3 rounds)	50	10.0	0 (0%)	0 (0%)	50 (100%)
3.	Variable changes – R2↑†, R3↓§	435‡	9.9	0 (0%)	336 (77%)	99 (23%)
11.	Variable changes – R2↔¶, R3↓§	46	8.7	0 (0%)	0 (0%)	46 (100%)
8.	Variable changes – R2↓§, R3↔¶	320	8.4	0 (0%)	301 (94%)	19 (6%)
10.	Variable changes – R2↔¶, R3↑† (positive change, below threshold)	367	7.6	0 (0%)	347 (95%)	20 (5%)
12.	No change (FIT values the same across all rounds)	2,773‡	4.7	2,759 (99.5%)	0 (0%)	14 (0.5%)

*A change is defined as the absolute value of the difference in FIT values between rounds being ≥4 µg/g (20 ng/ml). The positivity threshold is defined as 40 µg/g (200 ng/ml). †↑ Increase in FIT value from previous round. ‡Six participants were diagnosed with colorectal cancer: two in group 1, one in group 2 and, one in group 3 and two in group 12. §↓ Decrease in FIT value from previous round. ¶↔ No change in FIT value between rounds. ACN=advanced colorectal neoplasia. FIT=faecal immunochemical test. R2=round 2. R3=round 3.

Supplementary Table 2

Supplementary table 2. Detection of advanced colorectal neoplasia by serial changes groups, with serial changes defined by two different definitions (n=4,412).

	Type of change	Definition of change	Total N=4412 (%)	No ACN detected n=4100) (%)	ACN detected n=312 % (95% CI)
**1.**	**Serial increase above threshold (R2↑* – positive change, below threshold†), (R3↑* – positive change, above threshold)**	>0 ng/ml	51 (1.2)	35 (68.6)	16 31.4 (19.1 to 45.9)
		≥20 ng/ml	46 (1.0)	31 (67.4)	15 32.6 (19.5 to 48.0)
**2.**	**Serial increase below threshold (R2↑* – positive change, below threshold†), (R3↑* – positive change, below threshold)**	>0 ng/ml	103 (2.3)	86 (83.5)	17 16.5 (9.9 to 25.1)
		≥20 ng/ml	58 (1.3)	48 (82.8)	10 17.2 (8.6 to 29.4)
**3.**	**Variable changes – R2↑*, R3↓‡**	>0 ng/ml	475 (10.8)	428 (90.1)	47 9.9 (7.4 to 12.9)
		≥20 ng/ml	435 (9.9)	392 (90.1)	43 9.9 (7.2 to 13.1)
**4.**	**Variable changes – R2↑*, R3↔§**	>0 ng/ml	5 (0.1)	4 (80.0)	1 20.0 (0.5 to 71.6)
		≥20 ng/ml	59 (1.3)	52 (88.1)	7 11.9 (4.9 to 22.9)
**5.**	**Variable changes – R2↓‡, R3↑* (positive change, above threshold†)**	>0 ng/ml	47 (1.0)	35 (74.5)	12 25.5 (13.9 to 40.3)
		≥20 ng/ml	41 (0.9)	30 (73.2)	11 26.8 (14.2 to 43.0)
**6.**	**Variable changes – R2↓‡, R3↑* (positive change, below threshold†)**	>0 ng/ml	152 (3.5)	126 (82.9)	26 17.1 (11.5 to 24.0)
		≥20 ng/ml	126 (2.9)	105 (83.3)	21 16.7 (10.6 to 24.3)
**7.**	**Serial decrease↓‡ (serial decrease)**	>0 ng/ml	87 (2.0)	78 (89.7)	9 10.3 (4.8 to 18.7)
		≥20 ng/ml	50 (1.1)	45 (90.0)	5

					10.0 (3.3 to 21.8)
**8.**	**Variable changes – R2↓‡, R3↔§**	>0 ng/ml	302 (6.8)	277 (91.7)	25 8.3 (5.4 to 12.0)
		≥20 ng/ml	320 (7.3)	293 (91.6)	27 8.4 (5.6 to 12.0)
**9.**	**Variable changes – R2↔§, R3↑* (positive change, above threshold†)**	>0 ng/ml	80 (1.8)	71 (88.7)	9 11.3 (5.3 to 20.3)
		≥20 ng/ml	91 (2.0)	80 (87.9)	11 12.1 (6.2 to 20.6)
**10.**	**Variable changes – R2↔§, R3↑* (positive change, below threshold†)**	>0 ng/ml	348 (7.9)	325 (93.4)	23 6.6 (4.2 to 9.8)
		≥20 ng/ml	367 (8.3)	339 (92.4)	28 7.6 (5.1 to 10.8)
**11.**	**Variable changes – R2↔§, R3↓‡**	>0 ng/ml	3 (0.1)	3 (100)	0 0 (0 to 70.8)¶
		≥20 ng/ml	46 (1.0)	42 (91.3)	4 8.7 (2.4 to 20.8)
**12.**	**No change ↔§ (FIT values the same across all rounds)**	>0 ng/ml	2759 (62.5)	2632 (95.4)	127 4.6 (3.9 to 5.5)
		≥20 ng/ml	2773 (62.9)	2643 (95.3)	130 4.7 (3.9 to 5.5)

*↑ Increase in FIT value from previous round. †The positivity threshold is defined as 200 ng/ml. ‡↓ Decrease in FIT value from previous round. §↔ No change in FIT value between rounds. ¶One sided, 97.5% CI.
ACN=advanced colorectal neoplasia. FIT=faecal immunochemical test.

Sensitivity analysis investigating our definition of a ‘change’ between rounds. The table shows results when a ‘change’ between rounds was defined as i) >0 ng/ml or ii) ≥20 ng/ml. The difference in FIT result from round one to round two is represented by (R2) and the difference between round two and round three is represented by (R3).
